# Tumor-specific cytotoxicity of pyrazole-based chalcone derivatives in human oral squamous cell carcinoma cell lines

**DOI:** 10.55730/1300-0152.2773

**Published:** 2025-09-10

**Authors:** Mehtap TUĞRAK SAKARYA, Halise İnci GÜL, Hiroshi SAKAGAMI, Junko NAGAI, Yoshihiro UESAWA, Kenjiro BANDOW

**Affiliations:** 1 Department of Pharmaceutical Chemistry, Faculty of Pharmacy, Tokat Gaziosmanpaşa University, Tokat, Turkiye; 2 Department of Pharmaceutical Chemistry, Faculty of Pharmacy, Atatürk University, Erzurum, Turkiye; 3 Meikai University Research Institute of Odontology (M-RIO), Saitama, Japan; 4 Department of Medical Molecular Informatics, Meiji Pharmaceutical University, Tokyo, Japan; 5 Division of Biochemistry, Meikai University School of Dentistry, Saitama, Japan

**Keywords:** Pyrazole, QSAR analysis, ADMET/drug-likeness, tumor selectivity, cell cycle analysis, pyrazole-chalcone hybrids

## Abstract

**Background:**

Pyrazole-based chalcone hybrids are notable in medicinal chemistry for their potential biological activity, although their tumor-specific cytotoxicity and mechanisms remain unknown in OSCC cells. This first study of pyrazole-chalcone hybrids in OSCC cells explores the tumor-selective cytotoxic effects and underlying cell death mechanisms triggered by a series of 10 newly synthesized pyrazole-based compounds (**MS1MS10**) in OSCC cell lines.

**Material and methods:**

The cytotoxic effects of the compounds were assessed using the MTT assay on four human OSCC cell lines and three types of normal human oral cells. The tumor-selectivity index (TS) and potency-selectivity expression (PSE) were calculated, and active compounds were subjected to cell cycle analysis. For QSAR modeling, 3096 descriptors comprising physicochemical, structural, and quantum chemical features were created using the most energetically advantageous conformations found through CORINA optimization.

**Results:**

According to the obtained results, the compounds **MS4** (PSE = 1443.6, TS = 71.2), **MS7** (PSE > 15,304.5, TS > 247.4), and **MS8** (PSE > 7141.4, TS > 169.0) showed the highest TS and PSE values, comparable to those of doxorubicin and 5-FU. The cytotoxic compounds **MS7** and **MS8**, as well as the cytostatic compound **MS4**, significantly (p < 0.05) increased the cell population in the S and G2/M phases while decreasing the population in the G1 phase. Notably, no significant accumulation was detected in the sub-G1 phase, indicating the absence of DNA fragmentation-associated apoptosis. QSAR analysis suggests the importance of 3D structure and lipophilicity in TS expression, while ADMET analysis further revealed the drug-likeness properties of the active compounds. The obtained information is expected to contribute significantly to the literature on the design and development of new compounds.

**Conclusion:**

This study demonstrates the potent tumor-specific cytotoxic and cytostatic effects of pyrazole-based chalcone hybrids on OSCC cell lines, offering valuable insights for targeted anticancer drug development.

## 1. Introduction

Cancer is a complex illness caused by a mix of causative and predisposing variables and can occur at any time and under unfavorable conditions in susceptible individuals. Mortality from malignant neoplasms is one of the leading causes of death worldwide, making it a serious public health issue. Prostate cancer is more common in men and breast cancer is more common in women, while lung, bronchus, colon, and rectal cancers show dominance in neither sex (Jemal et al., 2007). Approximately 3% of malignant tumors begin in the oral cavity.

Squamous cell carcinoma (SCC) is a type of cancer that can affect several organs, including the skin, lips, mouth, esophagus, urinary bladder, prostate, lungs, vagina, and cervix. This cancerous tumor of the squamous epithelium has the potential to spread to other organs and metastasize. Oral cancer surgery can cause facial deformations, making it one of the most disfiguring cancers. The prognosis of these illnesses is determined by the size, infiltration, and location of the lesion, the presence or absence of metastasis, and, to some extent, the tumor’s differentiation (Esteban et al., 1998). Oral squamous cell carcinoma (OSCC) is the fifth most common cancer worldwide, and the number of cases is steadily increasing in emerging countries. Despite concerted efforts to enhance therapeutic techniques, 5-year survival rates for people with advanced-stage OSCC remain dismally low. OSCC, like other types of cancer, is a hereditary illness that results from differentiation loss and can be caused by a drop in apoptotic capacity and immunity (Vogelstein and Kinzler, 2004).

Many classes of potent anticancer drugs, including traditional anticancer chemotherapeutic agents, are currently available on the market. However, the majority of these drugs lack specificity and selectivity, leading to such problems as frequent severe side effects that reduce their overall therapeutic indices. This can be attributed to the presence of more than 100 different types of cancer, each of which has a diagnosis and requires a different course of treatment. Meeting the need for the development and identification of new small compounds with strong and specific anticancer properties continues to be a significant challenge for medicinal chemists (El-Adl et al., 2021 ).

**Figure 1:**
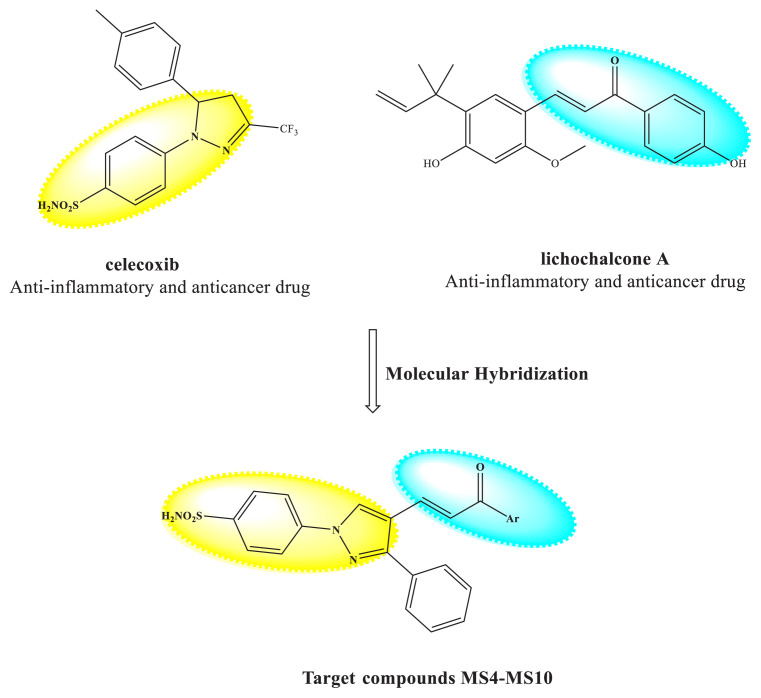
Structures of celecoxib and lichochalcone A, and rationale design of the target pyrazole/chalcone hybrids (**MS4-MS10**).

Celecoxib (Figure 1) is a nonsteroidal anti-inflammatory drug (NSAID) containing a 1,5-diaryl substituted pyrazole compound that selectively inhibits COX-2. It is commonly used for the treatment of several inflammatory illnesses, including osteoarthritis and rheumatoid arthritis. Furthermore, celecoxib has shown powerful anticancer or chemopreventive effects in vitro and in vivo (Sobolewski et al., 2010), and in patients. Celecoxib is expected to have anticancer properties across a wide range of malignancies, including colorectal, breast, and lung cancer. Its actions include cell growth inhibition, apoptotic cell death induction, immunological regulation, tumor microenvironmental manipulation, and antiangiogenic effects (Sobolewski et al., 2010). Celecoxib has also been investigated for the treatment of malignancies with COX-2 overexpression, such as breast cancer (Sobolewski et al., 2010). There have been several studies to date investigating the effects of celecoxib on different cancer cell lines and their sensitivity to celecoxib alone or in combination with other anticancer drugs (Sobolewski et al., 2010). For example, El-Awady et al. studied the concentration-dependent effects of celecoxib on five cancer cell lines, including HeLa, HCT116, HepG2, MCF-7, and U251, and reported HeLa cells to be the most resistant (IC_50_ = 37.2 μM) and U251 cells to be the most sensitive (IC_50_ = 11.7 μM) (El-Awady et al., 2011). In another study, Zhu et al. devised a number of approaches to the systematic modification of celecoxib’s structure to maximize its apoptosis-inducing effect (Zhu et al., 2002). Their first technique involved adding different substituents to celecoxib’s terminal aromatic ring or replacing it with different ring systems; the second involved replacing the sulfonamide group of several apoptosis-inducing celecoxib derivatives with a carboxamide group; and the third involved replacing celecoxib’s heterocyclic ring structure. All substances were evaluated for their capacity to cause apoptosis in human prostate cancer PC-3 cells, and it was noted that the apoptosis-inducing action of some compounds in the study was dramatically reduced when the sulfonamide group was replaced with a carboxamide group. It was further noted that the apoptosis-inducing effect was eliminated through the substitution of the pyrazole ring with other heterocyclic systems, and the deletion of the trifluoromethyl moiety from celecoxib’s pyrazole ring (Zhu et al., 2002).

Nitrogen-based heterocycles have garnered a lot of attention in time due to their significant roles in the design, development, and discovery of new drugs. Pyrazole scaffolds stand out among the *N*-containing heterocyclic pharmacophores as an intriguing template for combinatorial and medicinal chemistry, attributable to their exceptional physiological characteristics and promising applications in medicinal chemistry (Ansari et al., 2017). Numerous biological effects have been associated with pyrazole and its synthetic analogs, such as 1,3-diphenyl pyrazole, including anti-diabetic, depressive, anticonvulsant, antibacterial, anticancer, analgesic, and anti-inflammatory properties, (Ansari et al., 2017). Our study group synthesized molecules that contained both a pyrazole ring and a sulfonamide residue in their structures in earlier research, after which, their anticancer effects against OSCC cell lines were investigated. In vitro activity tests revealed that such compounds could guide the identification of potential anticancer drug candidates, and the results were consistent with previous findings reported in the literature (Gul et al., 2016; Gul et al., 2018; Tuğrak et al., 2021 ).

The biological activities of chalcone derivatives have also drawn considerable attention (Zhuang et al., 2017), including their antitumor (lichochalcone A, Figure 1), analgesic, anti-inflammatory (lichochalcone A, Figure 1), antibacterial, and anticancer effects (Goyal et al., 2021; Liu et al., 2024 ). Many chalcone derivatives have been shown to be cytotoxic in a variety of cancer cell lines via distinct signaling pathways (Tugrak et al., 2017; Dabhade et al., 2024 ).

**Figure 2:**
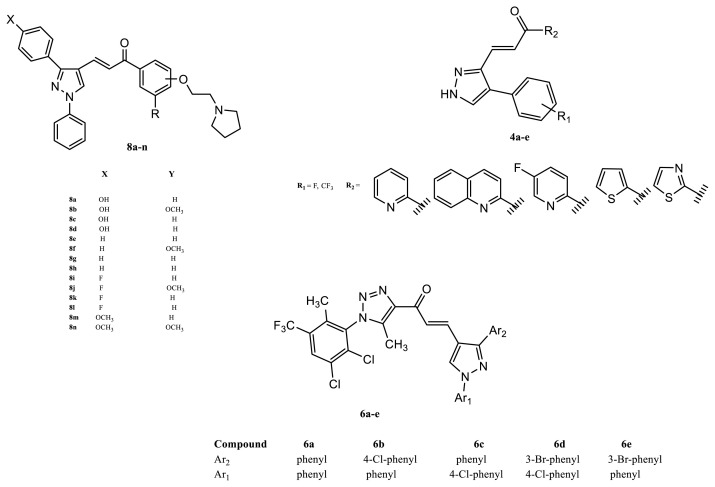
Chalcone-pyrazole derivatives have been the subject of considerable investigation in earlier research. **Reagent and conditions:** i: CH_3_COOH, Ethanol, Reflux, 19h, ii: POCl_3_, DMF, 21–23h, iii: NaOH, THF, iv: Appropriate acetophenone, NaOH, Methanol Ar = Phenyl (**MS4**), 4-methylphenyl (**MS5**), 4-methoxyphenyl (**MS6**), 2,4,5-trimethoxyphenyl (**MS7**), 3,4,5-trimethoxyphenyl (**MS8**), 3,4-dimetoxyphenyl (**MS9**), thiophenone-2-yl (**MS10**)

Pyrazole chalcone derivatives have emerged as a highly promising class of compounds with significant cytotoxic activity against a wide range of malignancies. Many studies to date have contributed significantly to the development of pyrazole chalcones (8a–n) (Dabhade et al., 2024). Bhat et al. developed a novel series of 1,3-diphenyl-pyrazole-chalcones (6a–e), the majority of which exhibited strong antiproliferative action against BC cell lines (Bhat et al., 2016). Furthermore, Rai et al. successfully synthesized novel 3-phenyl-4-alkyl-pyrazole-chalcone derivatives (4a–e) via strategic conjugation with various heterocycles and tested their anticancer activity on the MCF-7 cell line. Among their results, derivative 4c, featuring a 5-fluoropyridin-2-yl-pyrazole-chalcone structure, outperformed doxorubicin with a half-maximal inhibitory concentration (IC_50_) of 0.047 μM (Rai et al., 2015) (Figure 2). This study investigating the molecular hybridization of two bioactive entities in a single molecule, aims to create a novel series of pyrazole/chalcone hybrids by molecularly hybridizing the chalcone moiety and celecoxib drug (Figure 1), motivated by the promising anticancer activity of chalcone and pyrazole derivatives (Qin et al., 2015; Bukhari 2022).

OSCC is among the most common malignancies of the head and neck. While several chemotherapeutics are available on the market, they come with numerous side effects (Sugita et al., 2020). There is, therefore, a need for new anticancer medications that are highly selective against cancer cells while causing minimal or no harm to healthy cells. With this in mind, we carried out a series of studies to identify pyrazole-chalcone hybrid compounds appropriate for the treatment of oral cancer, adopting an in vitro evaluation system with human OSCC cell lines (Ca9-22, HSC-2, HSC-3, and HSC-4) and human normal oral cells (gingival fibroblast, HGF; periodontal ligament fibroblast, HPLF; pulp cell, HPC). The collected data were then subjected to a quantitative structure-activity relationship (QSAR) analysis.

## 2. Materials and methods

### 2.1. Chemistry

The structures of the **MS1–10** compounds were confirmed using ^1^H NMR (400 MHz), ^13^C NMR (100 MHz) with DMSO-*d_6_* as a solvent, and high-resolution mass spectrometry (HRMS). NMR data were collected using a Varian Mercury Plus spectrometer, and HRMS data were obtained with a Shimadzu mass spectrometer, operated in both positive and negative electrospray ionization (ESI) modes. Shimadzu’s LC-MS Solution software was used for data analysis. Melting points were measured with an Electrothermal 9100/IA9100 instrument and are reported without correction. Reaction progress was monitored through thin-layer chromatography (TLC) using silica gel 60 HF254 and a dichloromethane:methanol (4.5:0.5) solvent system.

#### 2.1.1. Synthesis of (E)-4-(2-(1-phenylethylidene)hydrazinyl)benzenesulfonamide, Compound MS1

**Figure 3:**
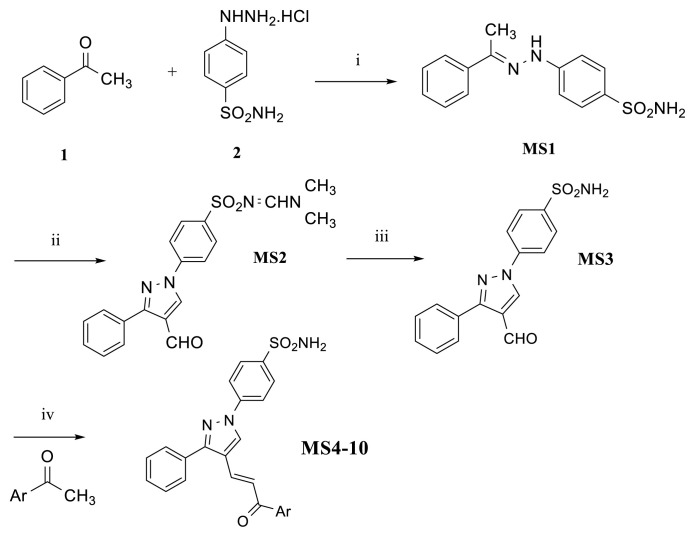
Synthesis of targeted **MS1–10** compounds.

Acetic acid (0.4 mmol) was added to a stirred solution of acetophenone **1** (8.3 mmol) and 4-hydrazinobenzenesulfonamide hydrochloride **2** (9.9 mmol) in ethanol, and the mixture was refluxed for 19 h. After the completion of the reaction, monitored by TLC, the mixture was allowed to stand at room temperature for 24 h. The separated solid product was filtered, washed with ethanol, dried, and crystallized from ethanol. Compound **MS1**: white solid; yield: 66%; m.p.: 257–258 °C; ^1^H NMR (DMSO-*d*_6_) δ, ppm: 9.73 (s, 1H, NH), 7.83 (d, *J* = 8.6 Hz, 2H, benzenesulfonamide), 7.68 (d, *J* = 8.8 Hz, 2H, benzenesulfonamide), 7.44–7.40 (m, 2H, Ar-H), 7.36–7.32 (m, 3H, Ar-H), 7.09 (s, 2H, NH_2_), 2.30 (s, 3H, CH_3_); HRMS (ESI-MS), m/z Calc.: 290.0958 C_14_H_15_N_3_O_2_S [M+H]^+^; Found: 290.0949 (Figure 3).

#### 2.1.2. Synthesis of *N’*-(4-(4-formyl-3-phenyl-1*H*-pyrazol-1-yl)phenyl)sulfonyl)-*N,N-*dimethylformimidamide, Compound MS2

Phosphoryl chloride (POCl_3_, 10 drops) was added dropwise to a solution of dimethylformamide (DMF) (0.188 mol) at room temperature under continuous stirring for 15–20 min. Hydrazone (compound **MS1**, 0.00925 mol) was added to the mixture and stirred for a few minutes, followed by refluxing at 70–80 °C for 21–23 h. The mixture was cooled and poured into the minimum amount of crushed ice, and then neutralized with a saturated solution of NaHCO_3_ to pH 7, filtered, dried, and recrystallized from ethanol. Compound **MS2**: Dark cream solid; yield: 45%; m.p.: 154–156 °C; ^1^H NMR (DMSO-*d*_6_) δ, ppm:10.01 (s, 1H, CHO), 9.45 (s, 1H, pyrazole-H), 8.28 (s, 1H, N=CH), 8.15 (d, *J* = 8.7 Hz, 2H, Ar-H), 7.98–7.93 (m, 4H, benzenesulfonamide), 7.55–7.52 (m, 3H, Ar-H), 3.18 (s, 3H, −N(CH_3_)_2_), 2.94 (s, 3H, −N(CH_3_)_2_); HRMS (ESI-MS), m/z Calc.: 383.1172 C_19_H_18_N_4_O_3_S [M+H]^+^; Found: 383.1171 (Figure 3).

#### 2.1.3. Synthesis of 4-(4-formyl-3-phenyl-1*H*-pyrazol-1-yl) benzenesulfonamide, Compound MS3

A stirred solution of NaOH (23.56 mmol) in methanol (20 mL) was added to *N*-protected formylpyrazole (**MS2**, 7.85 mmol). Tetrahydrofuran (THF, 60 mL) was then added to the mixture until a clear homogeneous solution was obtained. The mixture was stirred for 14 h and then poured into ice-cold water and neutralized with dilute hydrochloric acid (10%). The solid was separated out, filtered, washed with an excess of cold water, dried, and recrystallized from ethanol. Compound **MS3**: Light orange solid; yield: 38%; m.p.: 186–189 °C; ^1^H NMR (DMSO-*d*_6_, δ, ppm): 10.12 (s, 1H, CHO), 9.46 (s, 1H, pyrazole-H), 8.22 (d, *J* = 8.8, 2H, benzenesulfonamide), 8.01 (dd, *J* = 8.8, 2.2 Hz, 2H, benzenesulfonamide), 7.66–7.94 (m, 2H, Ar-H), 7.57–7.51 (m, 3H, Ar-H); ^13^C NMR (DMSO-*d*_6_, δ, ppm): 192.7, 157.4, 153.2, 142.8, 140.6, 135.4, 130.9, 129.4, 128.7, 128.6, 127.3, 122.6, 119.4; HRMS (ESI-MS), m/z Calc.: 328.0750 C_16_H_13_N_3_O_3_S [M+H]^+^; Found: 328.0754 (Figure 3).

#### 2.1.4. General synthesis method of (*E*)-4-(4-(3-oxo-3-(aryl)prop-1-en-1-yl)-3-phenyl-1*H*-pyrazol-1-yl)benzenesulfonamide, Compounds MS4-MS10

The appropriate acetophenone (6.7 mmol) was added to an ice-cold stirred solution of NaOH (18.0 mmol) in MeOH (30 mL) and the reaction mixture was stirred for 10 min at room temperature. This mixture was added to the appropriate 4-formylpyrazole (**MS3**, 5.6 mmol), then left to reach room temperature and stirred overnight. Upon completion of the reaction, the mixture was poured into ice-cold water and neutralized with dilute HCl to obtain a solid. The solid was filtered and washed with an excess of cold water, dried, and recrystallized from ethanol to afford chalcones **MS4–MS10** (Figure 3) (Tugrak et al., 2021).

##### (*E*)-4-(4-(3-Oxo-3-phenylprop-1-en-1-yl)-3-phenyl-1*H*-pyrazol-1 yl) benzenesulfonamide (MS4)

Yield 30%; m.p.: 276–278 °C. ^1^H NMR (DMSO-*d*_6_) δ, ppm: 9.53 (s, 1H, pyrazole-H), 8.13 (d, *J* = 8.8 Hz, 2H, Ar-H), 8.07 (d, *J* = 7.3 Hz, 2H, Ar-H), 8.02 (d, *J* = 8.7 Hz, 2H, Ar-H), 7.86 (d, *J* = 15.5 Hz, 1H, =CH), 7.69–7.65 (m, 5H, Ar-H, =CH−), 7.60–7.51 (m, 4H, Ar-H), 7.48 (s, 2H, SO_2_NH_2_). ^13^C NMR (DMSO-*d*_6_, 100 MHz, ppm) 193.9, 158.8, 147.5, 146.2, 142.8, 139.1, 138.4, 136.8, 134.6, 134.22, 134.17, 134.1, 133.7, 133.4, 132.7, 127.2, 123.9, 123.7. HRMS (ESI-MS), m/z Calc.: 430.1220 C_24_H_19_N_4_O_3_S [M+H]^+^; Found: 430.1217.

##### (*E*)-4-(4-(3-Oxo-3-(*p*-tolyl)prop-1-en-1-yl)-3-phenyl-1*H*-pyrazol-1-yl)benzenesulfonamide (MS5)

Yield 44%; m.p.: 306–308 °C. ^1^H NMR (DMSO-*d*_6_) δ, ppm: 9.52 (s, 1H, pyrazole-H), 8.16–8.13 (m, 2H, Ar-H), 8.06–7.98 (m, 4H, Ar-H), 7.86 (d, *J* = 15.4 Hz, 1H, =CH), 7.69–7.65 (m, 2H, Ar-H), 7.59–7.50 (m, 6H, Ar-H, CH=CH, SO_2_NH), 7.38 (d, *J* = 7.7 Hz, 2H, Ar-H), 2.40 (s, 3H, CH_3_). ^13^C NMR (DMSO-*d*_6_, 100 MHz, ppm) 188.0, 153.5, 143.6, 142.2, 140.9, 135.0, 133.3, 131.6, 129.3, 129.2, 128.9, 128.4, 128.3, 127.4, 121.9, 118.6, 118.5, 21.1. HRMS (ESI-MS), m/z Calc.: 444.1376 C_25_H_21_N_4_O_3_S [M+H]^+^; Found: 444.1360.

##### (*E*)-4-(4-(3-(4-Methoxyphenyl)-3-oxoprop-1-en-1-yl)-3-phenyl-1*H*-pyrazol-1-yl)benzenesulfonamide (MS6)

Yield 25%; m.p.: 244–246 °C; 226–228°C. ^1^H NMR (DMSO-*d*_6_) δ, ppm: 9.51 (s, 1H, pyrazole-H), 8.16–8.08 (m, 2H, Ar-H), 8.05–8.03 (m, 4H, Ar-H), 7.88 (d, *J* = 15.4 Hz, 1H, =CH), 7.60–7.50 (m, 6H, Ar-H, −CH_2_ SO_2_NH), 7.11 (d, *J* = 8.8 Hz, 2H, Ar-H), 3.87 (s, 3H, OCH_3_). ^13^C NMR (DMSO-*d*_6_, 100 MHz, ppm) 186.8, 163.2, 153.4, 142.1, 140.9, 132.8, 131.6, 130.5, 130.4, 129.1, 128.9, 128.4, 128.3, 127.4, 121.9, 118.6, 114.0, 55.6. HRMS (ESI-MS), m/z Calc.: 460.1326 C_25_H_21_N_4_O_4_S [M+H]^+^; Found: 460.1329.

##### (*E*)-4-(4-(3-Oxo-3-(2,4,5-trimethoxyphenyl)prop-1-en-1-yl)-3-phenyl-1*H*-pyrazol-1-yl)benzenesulfonamide (*MS7*)

Yield 45%; m.p.: 245–246 °C. ^1^H NMR (DMSO-*d*_6_) δ, ppm: 9.30 (s, 1H, pyrazole-H), 8.17 (d, *J* = 8.7 Hz, 2H, Ar-H), 8.01 (d, *J* = 8.7 Hz, 2H, Ar-H), 7.68 (d, *J* = 7.2 Hz, Ar-H), 7.59–7.49 (m, 7H, Ar-H, −CH=, SO_2_NH), 7.02 (s, 1H, Ar-H), 3.95 (s, 1H, Ar-H), 3.89 (s, 6H, OCH_3_), 3.74 (s, OCH_3_). ^13^C NMR (DMSO-*d*_6_ 100 MHz, ppm) 186.8, 163.2, 153.4, 148.0, 147.8, 146.2, 143.2, 142.1, 140.9, 131.8, 131.6, 129.2, 128.4, 128.3, 127.4, 121.9, 118.7, 118.5, 113.5, 112.8, 97.8, 56.8, 55.9. HRMS (ESI-MS), m/z Calc.: 520.1537 C_27_H_25_N_4_O_5_S [M+H]^+^; Found: 520.1539.

##### (*E*)-4-(4-(3-Oxo-3-(3,4,5-trimethoxyphenyl)prop-1-en-1-yl)-3-phenyl-1*H*-pyrazol-1-yl)benzenesulfonamide (MS8)

Yield 34%; m.p.: 168–170 °C. ^1^H NMR (DMSO-*d*_6_) δ, ppm: 9.30 (s, 1H, pyrazole-H), 8.18 (d, *J* = 8.1 Hz, 2H, Ar-H), 8.01 (d, *J* = 8.2 Hz, 2H, Ar-H), 7.68–7.49 (m, 7H, Ar-H, =CH, SO_2_NH_2_), 7.21 (s, 1H, Ar-H), 6.79 (s, 1H, Ar-H), 3.90 (s, 6H, OCH_3_), 3.75 (s, 3H, OCH_3_).^13^C NMR (DMSO-*d*_6_, 100 MHz, ppm) 189.2, 154.9, 154.0, 153.8, 143.3, 142.6, 141.5, 132.3, 131.9, 129.5, 129.4, 129.3, 128.9, 127.8, 127.6, 119.7, 119.2, 118.9, 113.3, 98.4, 57.1, 56.4. HRMS (ESI-MS), m/z Calc.: 520.1537 C_27_H_25_N_4_O_5_S [M+H]^+^; Found: 520.1540.

##### (*E*)-4-(4-(3-(3,4-Dimethoxyphenyl)-3-oxoprop-1-en-1-yl)-3-phenyl-1*H*-pyrazol-1-yl)benzenesulfonamide (MS9)

Yield 31%; m.p.: 177–179 °C. ^1^H NMR (DMSO-*d*_6_) δ, ppm: 9.49 (s, 1H, pyrazole-H), 8.14 (d, *J* = 8.7 Hz, 2H, Ar-H), 8.04 (d, *J* = 8.4 Hz, 2H, Ar-H), 7.98 (s, 1H, Ar-H), 7.79–7.70 (m, 2H, Ar-H), 7.68–7.45 (m, 7H, Ar-H −CH=, SO_2_NH_2_), 7.30 (s, 1H, Ar-H, *J* = 8.4 Hz), 3.88 (s, 3H, OCH_3_), 3.85 (s, 3H, OCH_3_). ^13^C NMR (DMSO-*d*_6_, 100 MHz, ppm): 190.0, 185.9, 154.3, 148.8, 142.0, 140.2, 139.2, 130.4, 129.1, 128.9, 128.7, 128.5, 127.9, 127.4, 122.9, 121.9, 121.5, 118.6, 113.3, 55.6. HRMS (ESI-MS), m/z Calc.: 490.1431 C_26_H_23_N_4_O_5_S [M+H]^+^; Found: 490.1429.

##### (*E*)-4-(4-(3-Oxo-3-(thiophen-2-yl)prop-1-en-1-yl)-3-phenyl-1*H*-pyrazol-1-yl)benzenesulfonamide (MS10)

Yield 20%; m.p.: 281–282 °C. ^1^H NMR (DMSO-*d*_6_) δ, ppm: 9.50 (s, 1H, pyrazole-H), 8.16–8.13 (m, 2H, Ar-H), 8.00–7.94 (m, 4H, Ar-H), 7.76 (d, *J* = 15.4 Hz, 1H, =CH), 7.71–6.50 (m, Ar-H), 7.61–7.50 (m, 6H, Ar-H, −CH=, SO_2_NH_2_), 7.37 (d, 1H, Ar-H, *J* = 5.3 Hz).^13^C NMR (DMSO-*d*_6_, 100 MHz, ppm) 181.1, 153.5, 143.5, 142.2, 140.9, 135.5, 132.7, 132.1, 131.5, 129.2, 128.99, 128.23, 128.2, 128.0, 127.5, 121.9, 118.7, 118.3. HRMS (ESI-MS), m/z Calc.: 436.0784 C_22_H_17_N_4_O_3_S_2_ [M+H]^+^; Found: 436.0782.

### 2.2. Materials

The following chemicals and reagents were purchased from their respective listed companies: Dulbecco’s modified Eagle’s medium (DMEM) from GIBCO BRL (Grand Island, NY, USA); fetal bovine serum, 3-(4,5-dimethylthiazol-2-yl)-2,5-diphenyltetrazolium bromide (MTT), doxorubicin, ribonuclease (RNase) A from Sigma-Aldrich Inc. (St. Louis, MO, USA); propidium iodide (PI), dimethyl sulfoxide (DMSO), actinomycin D, and 4% paraformaldehyde phosphate buffer solution from Wako Pure Chem. Ind. (Osaka, Japan); 5-fluorouracil (5-FU) from Kyowa (Tokyo, Japan); Nonidet-40 (NP-40) from Nakalai Tesque Inc. (Kyoto, Japan); culture plastic dishes and 96-well plates from Techno Plastic Products AG (Trasadingen, Switzerland).

### 2.3. Cell culture and assay for cytotoxic activity

Human OSCC cell lines (Ca9-22, HSC-2, HSC-3, HSC-4) and three types of human normal oral cells—gingival fibroblast (HGF), periodontal ligament fibroblast (HPLF), and pulp cells (HPC)—established from the first premolar extracted tooth in the lower jaw and periodontal tissues of a 12-year-old girl) were cultured in DMEM supplemented with 10% heat-inactivated FBS, 100 U/mL, and antibiotics under a humidified 5% CO_2_ atmosphere. The viable cell number was determined in triplicate by the MTT method, following the approach adopted in previous studies (Nagai et al., 2018; Shi et al., 2018 ). Briefly, the cells were incubated for 2 h with 0.2 mg/mL of MTT, and the formed formazan was dissolved with 0.1 mL of DMSO to determine the relative viable cell number at 560 nm.

### 2.4. Cell-cycle analysis

Cell-cycle analysis was performed following the process described in the literature (Kantoh et al., 2010; Nagai et al., 2018; Shi et al., 2018), details of which are presented in a supplementary file. Briefly, cells (approximately 10^6^ in number) were fixed with paraformaldehyde in PBS(−) and treated with RNase A. After staining with propidium iodide in the presence of 0.01% Nonidet-40 to prevent cell aggregation, the cells were filtered through Falcon cell strainers and then subjected to cell sorting, analyzed with Cell Sorter Software (version 2.1.2) (SONY Imaging Products and Solution Inc., Kanagawa, Japan).

### 2.5. Statistical treatment

The CC_50_ values were expressed as mean ± S.D. from triplicate assays. The relationships among cytotoxicity, tumor specificity index, and chemical descriptors were investigated with simple regression analyses using JMP Pro (version 14.3.0) (SAS Institute Inc., Cary, NC, USA). The significance level was set at p < 0.05^1^. For multiple comparisons, a one-way analysis of variance (ANOVA) followed by Bonferroni’s post hoc test was performed (IBM SPSS Statistics (version 27.0)). A *p*-value of < 0.05 was considered statistically significant. Details of the computational analysis details are presented as a supplementary file (Uesawa et al., 2015).

## 3. Results and discussion

### 3.1. Cytotoxicity against OSCCs

**Figure 4:**
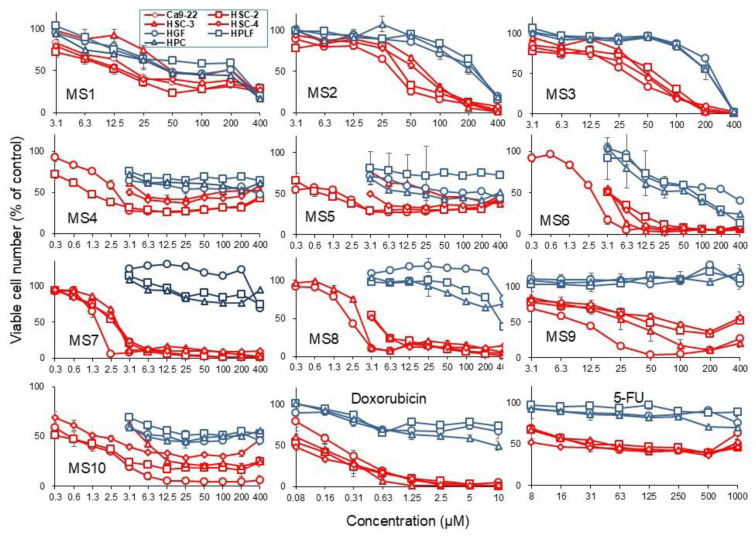
The cytotoxic effects of **MS1–10** compounds, doxorubicin (DXR), and 5-FU were evaluated against four human OSCC cell lines: Ca9-22, HSC-2, HSC-3, and HSC-4. Additionally, the effects on human normal oral cells, including human gingival fibroblast (HGF), human periodontal ligament fibroblast (HPLF), and human pulp cells (HPC), were assessed. Cells were incubated for 48 h with the indicated concentrations of **MS4**, **MS7**, or **MS8**, or without (for control). Cell viability was determined by the MTT method and expressed as a percentage of the control. Each value represents the mean ± S.D. of the triplicate.

Compounds **MS1–10** were tested to assess their cytotoxicity against human OSCC cell lines (Ca9-22, HSC-2, HSC-3, and HSC-4) and human normal oral cells (gingival fibroblast, HGF; periodontal ligament fibroblast, HPLF; pulp cell, HPC). Dose-response curves of compounds **MS1–10** and the reference compounds doxorubicin (DXR) and 5-fluorouracil (5-FU) are presented in Figure 4. Compounds **MS1–10**, along with the two reference compounds DXR and 5-FU, inhibited the growth of the four OSCC cell lines (indicated in red) more potently than that of normal oral cells (in blue), as shown in Figure 4, suggesting a tumor-specific action. The compounds exhibited two different types of growth inhibition: **MS1**, **MS4**, **MS5**, and 5-FU induced cytostatic effects on the OSCC cell lines (without complete cytotoxicity), while **MS2**, **MS3**, **MS6**, **MS7**, **MS8**, and DXR induced cytotoxic effects, (leading to complete cell death). **MS9** and **MS10** induced mixed effects, depending on the target cells (Figure 4).

Table 1 lists the 50% cytotoxic concentrations (CC_50_) of the compounds, determined from the dose-response curve and tumor-selectivity index [TS = mean CC_50_ (normal cells) / mean CC_50_ (OSCC cell lines)]. **MS7** showed the highest TS value (TS > 148.7), followed by **MS8** (> 113.5), **MS4** (> 103.7), DXR (> 54.9), 5-FU (> 28.6), and the remaining MS compounds (D/B in Table 1). Since HGF represents the normal cell type associated with the Ca9-22 OSCC cell line, both of which are derived from gingival tissues, TS values were calculated by dividing the average CC_50_ value for HGF cells by the CC_50_ value for Ca9-22 cells (C/A, Table 1). **MS7** was again found to show the highest TS value (TS > 247.4), followed by **MS8** (> 169.0), **MS5** (88.8), **MS6** (66.5), **MS4** (71.2), DXR (> 45.5), 5-FU (> 30.3) and the remaining MS compounds (C/A in Table 1).

To identify the most promising compounds in terms of potency and selective cytotoxicity, PSE values were computed by dividing the TS value by the cytotoxicity of tumor cells and multiplying the result by 100. When using four tumor cells and three normal cells, **MS7** showed the highest PSE value (PSE > 5531)(100xD/B^2^ in Table 1), followed by **MS8** (> 3389) > **MS4** (> 2800) > other MS compounds. **MS7**, **MS8**, and **MS4** showed higher TS values than 5-FU (> 82), but not DXR (> 32,929), which showed the highest cytotoxicity against OSCC cell lines (Table 1). When using Ca9-22 and HGF, both derived from gingiva, **MS7** again exhibited the highest PSE value (PSE > 15,305)(100xC/A^2^ in Table 1), followed by **MS8** (> 7141), **MS5** (5426), **MS10** (4191), **MS6** (2115), **MS4** (> 1444), and the remaining MS compounds. **MS7**, **MS8**, and **MS4** exhibited higher TS values than 5-FU (> 92), but not DXR (> 20,661), which exhibited the highest cytotoxicity to Ca9-22 cells (Table 1).

A SAR analysis was performed based on the TS and PSE values of the compounds, revealing very weak tumor selectivity in **MS1**, **MS2**, and **MS3** (TS = 4.5–4.7, PSE = 8.5–17.2). The introduction of the ArCOCH_3_ group yielded seven pyrazole-based chalcone derivatives with the following substituents: phenyl (**MS4**), 4-methyl phenyl (**MS5**), 4-methoxyphenyl (**MS6**), 2,4,5-trimethoxyphenyl (**MS7**), 3,4,5-trimethoxyphenyl (**MS8**), 3,4-dimethoxyphenyl (**MS9**), and thiophenone-2-yl (**MS10**) (Figure 3). **MS4** exhibited highly elevated tumor selectivity (TS > 103.7, PSE > 2799.6). The addition of methoxy to theMS4 generally increased cytotoxicity towards Ca9-22, but not in **MS9**, which had 3,4-dimethoxyphenyl. The presence of three substituted methoxy groups in compounds **MS7** and **MS8** increased both cytotoxicity and tumor selectivity, while reducing the methoxy number to two in compound **MS9** led to a decrease in these values.

Among the methoxylated compounds, **MS7**, the leading compound, was 20.6 times more cytotoxic towards Ca9-22 than the 5-FU reference compound. Although bioisosteric substitution generally resulted in increased cytotoxicity, the thiophene ring (**MS10**) was less favorable than the phenyl derivative (**MS4**) in terms of its cytotoxicity, TS, and PSE values. When the cytotoxicity of the compounds in the series (**MS4–MS10**) against the HSC-2 cell line were evaluated, methoxylation led to a decrease in the cytotoxicity of the compounds.

Among the compounds with a methoxyl substituent group, **MS9**—again, a dimethoxy derivative—was found to be the least favorable compound in the series. Furthermore, the thiophene ring (**MS10**) was more favorable than the phenyl derivative (**MS4**) towards the HSC-2 cancer cell line. In the pyrazole/chalcone derivatives (**MS4–MS10**), cytotoxicity was increased in the methoxylated compounds **6**, **7**, and **8** by 1.5–2 times, while the compounds containing 2,5-dimethoxyphenyl were the weakest against the HSC-3 cancer cell line when compared to the unsubstituted phenyl derivative **MS4**. Compound **MS10**, which has a thiophene ring, was found to be almost as cytotoxic as its phenyl analog against HSC-3. When the cytotoxicity of the compounds was evaluated against the HSC-4 cancer cell line, all methoxylated derivatives were noted to have almost the same cytotoxic effect, other than compound **MS9**. Unlike in the other cell lines, bioisosteric replacement led to a significant increase in cytotoxicity in compound **MS10**.

This work demonstrated that several synthetic pyrazole-chalcone compounds, including **MS7** and **MS8**, had significant tumor selectivity and activity against OSCC cell lines in comparison to normal oral cells. In the larger context of cancer research, it is significant that prior studies have examined the benefit of structurally analogous pyrazole-chalcone compounds against diverse cancer cell lines, yielding supplementary data on their possible therapeutic applications.

In a study conducted by Dabhade et al., a series of pyrazole-chalcone derivative compounds were synthesized, with novel biphenyl-substituted pyrazole-chalcone derivatives bearing a pyrrolidine ring designed using a hybridization approach, and their effects on MCF-7 and MDA-MB-231 cells were evaluated using the NRU assay. Among the compounds, 8k, 8d, 8m, 8h, and 8f exhibited remarkably potent IC_50_ values of 0.17, 5.48, 8.13, 20.51, and 23.61 μM, respectively, against MCF-7 cells when compared with the positive controls raloxifene and tamoxifen. Furthermore, the most active compound, 8k [3-(3-(4-fluoro-phenyl)-1-phenyl-1*H*-pyrazol-4-yl)-1-(2-(2-(pyrrolidin-1-yl)-ethoxy)-phenyl)-chalcone], caused cell death via apoptosis and arrest in the G2/M phase of the cell cycle (Figure 2).

In another study, pyrazole-chalcone derivatives (6a**–e**, (2*E*)-1-{1-[2,3-dichloro-6-methyl-5-(trifluoromethyl)phenyl]-5-methyl-1*H*-1,2,3-triazol-4-yl}-3-(1,3-diaryl-1*H*-pyrazol-4-yl)prop-2-en-1-one) were prepared utilizing the *Claisen-Schmidt* condensation process and the produced compounds were tested in vitro for antibacterial, antioxidant, and anticancer activity. The majority of the synthesized compounds revealed broad-spectrum antibacterial and antioxidant actions, with some showing moderate to good anticancer activity against breast cancer cell lines. Overall, the reported study identified some promising precursors with potential antibacterial, antioxidant, and anticancer properties (Figure 2).

A study by Rai et al. presented details of their synthesis and characterization of novel heterocyclic pyrazole chalcone derivatives (4a–e). Pyrazole chalcones were synthesized by reacting pyrazole aldehydes with suitable aromatic ketones, and the newly synthesized compounds were characterized using spectroscopic methods, while their biological activities were evaluated in vitro using MCF-7 (human breast adenocarcinoma) and HeLa (human cervical tumor cells) cell lines. Among the compounds, 4c exhibited the greatest inhibition in human MCF-7 and HeLa cell lines. This was attributed to the presence of 4-fluoro-phenyl and 5-fluoro-pyridin moieties, which contributed to its superior action within the low micromolar to nanomolar concentration range. This molecule thus shows potential for advancement into a novel category of anticancer therapeutics (Figure 2).

The present study offers a more comprehensive analysis of cytotoxicity by investigating multiple human OSCC lines and various normal oral cell types. In this way, it differs from earlier studies on pyrazole-chalcone derivatives that primarily focused on breast and cervical cancer cell lines, and thereby enabling a thorough evaluation of tumor selectivity. The study includes tumor selectivity index (TS) and potency-selectivity expression (PSE) analysis, providing a deeper insight into the therapeutic spectrum of each compound, unlike earlier studies that reported only IC_50_ values and lacked a comprehensive selectivity assessment. Furthermore, the structure-activity relationship (SAR) revealed in the present study clearly demonstrates the impact of methoxylation and biosteric changes on cytotoxic potency and selectivity.

### 3.2. Different effects of MS4 and MS7/8 on cell cycle progression

**Figure 5:**
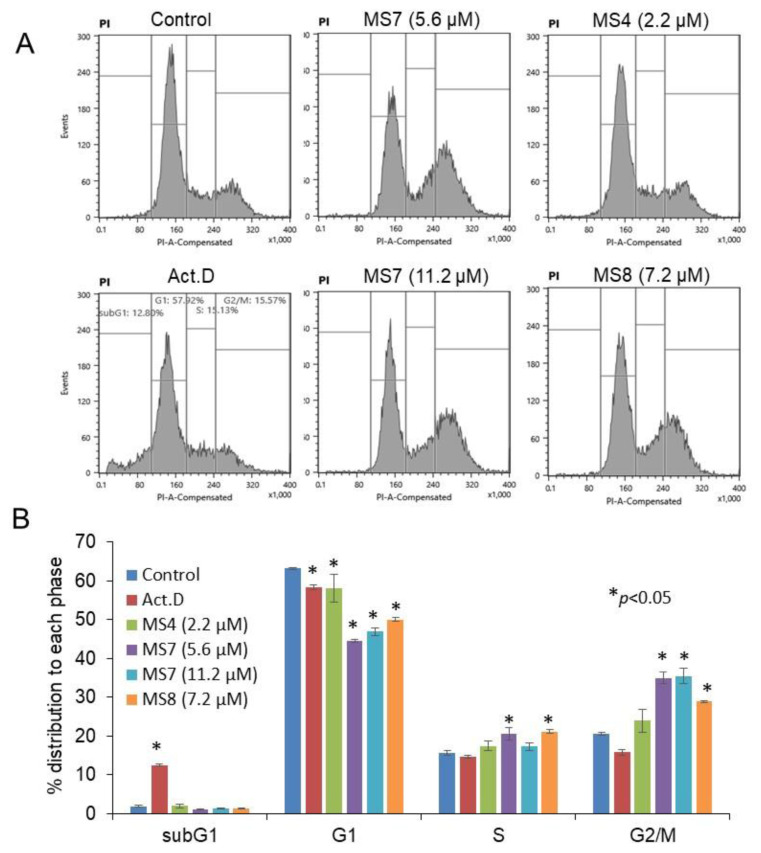
The effect of **MS4**, **7**, and **8** on cell cycle distribution in the OSCC cell line HSC-2. Cells were treated for 24 hours with the appropriate concentrations of compounds **MS4**, **7**, and **8** or actinomycin D (Act D) as a positive control, after which cell sorting was used to analyze cell-cycle distribution. Since the CC_50_ values of compounds **MS4**, **MS7**, and **MS8** against HSC-2 cells are 1.1, 2.8, and 3.6 μM, respectively, the cytotoxicity of these compounds is 3.3, 1.3, and 1-fold. Based on this information, the 2.2, 5.6, and 7.2 μM concentrations were selected. Only **MS7**, which has the highest tumor selectivity, was tested at two concentrations (5.6 and 11.2 μM), revealing that it has reached a plateau level of growth inhibition.

HSC-2 cells were incubated for 24 h with actinomycin D (Act.D) (positive control of apoptosis induction) or **MS4**, **7**, or **8**, or without treatment (control), and subjected to cell cycle analysis. As shown in Figure 5, the subG1 cell population was significantly increased in Act. D (1 μM) (p < 0.05) from 1.9 % to 12.5%. In contrast, no increase in the number of subG1 cell populations—composed of DNA fragments produced by either apoptosis or other types of cell death—was observed in **MS4** (2.2 μM), **MS7** (5.6, 11.2 μM), or **MS8** (7.2 μM) (2.0%, 1.0%, 1.3%, and 1.3%, respectively). **MS7** and **MS8**, which induced the inhibition of cytotoxic growth, significantly (p < 0.05) increased the share of S-phase cells (from 15.6% to 20.6% and 21.2%, respectively), and more prominently, the share of G2+M phase cells (from 20.5% to 35.0% and 35.4%, respectively), resulting in a significant decline in G1 phase cells (from 63.2% to 44.5% and 50.0%, respectively). On the other hand, **MS4**, which induced cytostatic growth inhibition, only slightly induced such changes in cell cycle distribution. The cell cycle analysis was performed only with HSC-2 cells; therefore, experiments with other OSCC cell lines should be conducted to support the generalization of our findings.

### 3.3. Computational analysis

**Figure 6:**
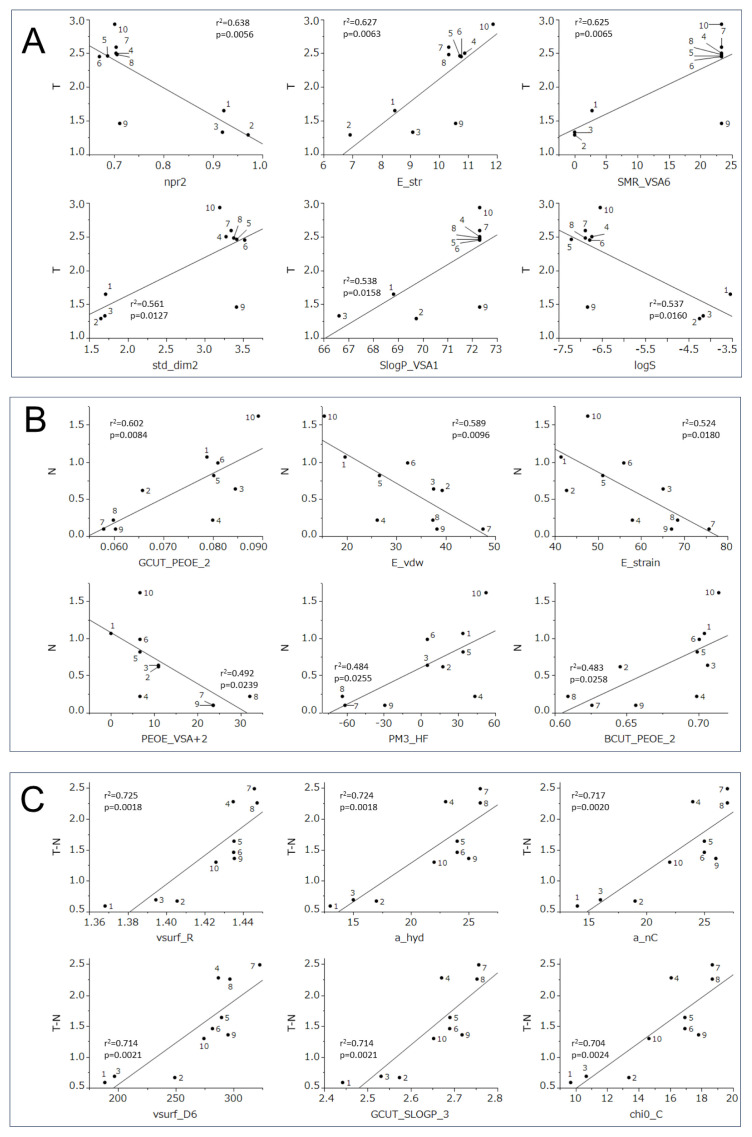
Top six chemical descriptors that showed higher correlation with cytotoxicity of 10 compounds [**MS1–10**] against OSCC cells (A), non-malignant cells (B), and with tumor-specificity (C). The mean negative log CC_50_ values against tumor cells (T) (A), normal cells (N)(B), and tumor-specificity (T-N) (C) were plotted. CC_50_: concentration of compound that reduced the viable cell number by 50%. The top six chemical descriptors are explained in Table 2.

A quantitative structure-activity relationship (QSAR) analysis of 10 MS compounds was performed to understand their cytotoxicity against normal and tumor cells. Since the 31, 17, and 141 descriptors were significantly correlated with cytotoxicity against OSCC (T), normal cells (N), and tumor-specificity (TS) (p < 0.05 and p < 0.005, respectively) (Supplementary Tables 1, 2, and 3), the top six chemical descriptors were selected for QSAR analysis (Figures 6, and Table 2).

The cytotoxicity of compounds **MS1–10** against human OSCC cell lines was correlated positively with E_str (bond stretch energy) (r^2^ = 0.627, p = 0.0063), SMR_VSA6 (topological shape) (r^2^ = 0.625, p = 0.0065), std_dim2 (3D shape) (r^2^ = 0.561, p = 0.0127) and SlogP_VSA1 (lipophilicity)(r^2^ = 0.538 p = 0.0158), and negatively with npr^2^ (3D shape and volume) (r^2^ = 0.638, p = 0.0056) and logS (lipophilicity) (r^2^ = 0.537, p = 0.0160) (Figure 6A).

The cytotoxicity of compounds **MS1–10** against human normal oral mesenchymal cells was correlated positively with GCUT_PEOE_2 (topological shape and partial charges) (r^2^ = 0.602 p = 0.0084), PM3_HF (heat of formation) (r^2^ = 0.484, p = 0.0255) and BCUT_PEOE_2 (topological shape and partial charges) (r^2^ = 0.483, p = 0.0258), and negatively with E_vdw (van der Waals energy) (r^2^ = 0.589, p = 0.0096), E_strain (local strain energy) (r^2^ = 0.524, p = 0.0180) and PEOE_VSA+2 (topological shape and partial charges) (r^2^ = 0.492, p = 0.0239) (Figure 6B).

The TS of compounds **MS1–10** was correlated positively with all vsurf_R (3D shape) (r^2^ = 0.725, p = 0.0018), a_hyd (number of hydrophobic atoms) (r^2^ = 0.724, p = 0.0018), a_nC (number of carbon atoms) (r^2^ = 0.717, p = 0.0020), vsurf_D6 (3D size and lipophilicity) (r^2^ = 0.714, p = 0.0021), GCUT_SLOGP_3 (topological shape) (r^2^ = 0.714, p = 0.0021) and chi0_C (topological shape) (r^2^ = 0.704, p = 0.0024) (Figure 6C).

QSAR analysis revealed the cytotoxicity of compounds **MS1–10** against tumor cell lines to be highly significantly correlated with bond stretch energy, topological shape, 3D shape, volume, and lipophilicity (Figure 6A). Furthermore, their tumor specificity was significantly correlated with 3D shape and size, number of hydrophobic atoms and carbon atoms, 3D sizes and lipophilicity, and topological shape (Figure 6C). Taken together, these data suggest a positive relationship between tumor selectivity and chemical descriptors related to 3D structure and lipophilicity.

The present QSAR analysis is an exploratory descriptor-activity correlation conducted on a small, focused compound set (n = 10; **MS1–10**), and so some limitations should be considered. A total of 3096 physicochemical and structural descriptors were calculated, although only the top six descriptors per endpoint with the highest correlation are reported (Figure 6, Table 2). The observed correlations (overall r^2^ = 0.483–0.725; all p < 0.05) should therefore be interpreted as hypothesis-generating trends rather than as a predictive model. Furthermore, given the small sample size, the possibility of chance correlations and the instability of feature ranking should be considered, along with the narrow applicability domain. The QSAR analysis in this study is intended to prioritize structural features—particularly 3D shape/size and lipophilicity—as design guidance, rather than for external prediction.

### 3.4. Druglikeness properties of lead compounds

To be effective, a powerful molecule must reach its target in the body at sufficient concentration and remain in a bioactive form long enough for the predicted biological activities to occur. In the early stages of drug development, the molecule’s absorption, distribution, metabolism, and excretion (ADME) must be assessed, although these ADME parameters can be evaluated independently with specific approaches. Previous studies have reported that estimating ADME early in the discovery phase can substantially reduce the rate of pharmacokinetic-related failure in the clinical phase. It will therefore be beneficial to begin assessing the compounds’ ADME properties in the initial stage. Table 3 shows the computed molecular weight (MW), partition coefficient (logP), hydrogen bond donor (HBD), hydrogen bond acceptor (HBA), molecular refractivity (MR), topological polar surface area (tPSA), gastrointestinal (GI), and blood-brain barrier (BBB) absorption values. Druglikeness assessments qualitatively examine the suitability of drugs for oral use in terms of bioavailability. Such assessments investigate the structural or physicochemical properties of compounds to ensure they are sufficiently developed to be considered as oral drug candidates. This approach is commonly employed in a variety of filters, including Lipinski RO5, Ghose, Veber, Egan, and Muegge, for the identification of molecules with a suitable pharmacokinetic profile and those with incompatible features. Druglikeness, physicochemical, and pharmacokinetic properties of the lead compounds in the present study were analyzed using SwissADME software (Daina et al, 2017), the results of which are presented in Table 4, along with their limitations (Sakarya et al., 2023; Yamali et al., 2023; Yamali et al., 2024)

With a few exceptions, the compounds fit the stated filters. The following parameters were calculated based on the study results (Table 3): molecular weight (289.35–519.57), number of H-bond donors (0–2), number of H-bond acceptors (4–8), and logP (lipophilicity, 1.73–3.82). Most of the compounds were found to be compatible with Lipinski’s RO5.

Although **MS7** and **MS8** do not entirely conform to the Ghose druglikeness filter, it should be remembered that such filters are empirical guides rather than absolute laws for lead selection. The Ghose filter helps to describe a typical drug-like chemical space; however, many effective leads and approved medications, particularly those with complex or innovative scaffolds, do not fit its parameters. Importantly, **MS7** and **MS8** have much higher tumor-selectivity indices and potency-selectivity expression than the other members in the series and the clinical reference 5-FU, demonstrating their biological advantages. As a result, notwithstanding their divergence from the Ghose criteria, these compounds can be considered intriguing candidates for future improvement in OSCC (Doak et al., 2014)

## 4. Conclusion

The present study has investigated the design and synthesis of new pyrazole-chalcone hybrids as potential anticancer drugs. Based on the findings of cell cycle research, cytotoxicity testing, and QSAR analysis, we concluded that pyrazole-based chalcone hybrids could be considered an impressive family of compounds. Of the 10 synthesized compounds, three—**MS4**, **MS7**, and **MS8**—demonstrated extraordinary potency against cancer cell lines with PSE and TS values of (PSE = 1443.6, TS = 71.2); (PSE = > 15,304.5, TS = > 247.4); and (PSE = >7141.4, TS = > 169.0), respectively. Compounds **MS4**, **MS7**, and **MS8** showed higher cytotoxicity against the cells used, and the dose-response study identified cytotoxic growth inhibition in both **MS7** and **MS8**, and cytostatic growth inhibition in **MS4**. Cell cycle data indicated that **MS4**, **7**, and **8** induced G2+M arrest. Further investigations of the expression of several apoptosis markers, such as Annexin V/PI staining or caspase activation, is needed to clarify whether G2+M accumulation leads to apoptosis induction. Furthermore, ADMET analysis revealed the druglikeness properties of the active compounds. Finally, bioassays showed that lead compounds **MS4**, **MS7**, and **MS8** hold promise as candidates for the development of a new type of anticancer drug. The information garnered in the present study may contribute significantly to the literature on the design and development of new compounds.

Based on the findings of the present study, **MS7**, as the most potent compound, may have the best-fitted 3D structure and/or lipophilicity to the target molecule. If such a promising candidate molecule is estimated through the signaling pathway analysis of various receptors, an even more potent compound could be revealed (Naitoh et al., 2022; Abe et al., 2023).

## Supplementary Information











## Figures and Tables

**Table 1:** Cytotoxic activity of 10 MSs against oral malignant and nonmalignant cells.

	CC_50_ (μM)														
	OSCC						Normal oral cells								
	Ca9-22	HSC-2	HSC-3	HSC-4	mean	SD	HGF	HPLF	HPC	mean	SD	TS		PSE	
	(A)				(B)		(C)			(D)		(D/B)	(C/A)	(D/B^2^)x100	(C/A^2^)x100
**MS1**	20.0	14.3	53.7	15.7	25.9	18.7	50.7	245.7	51.0	115.8	112.5	4.5	2.5	17.2	12.6
**MS2**	34.3	41.3	74.3	63.0	53.3	18.6	245.0	262.3	214.3	240.6	24.3	4.5	7.1	8.5	20.8
**MS3**	32.3	64.0	51.7	46.3	48.6	13.1	253.0	217.0	220.7	230.2	19.8	4.7	7.8	9.8	24.2
**MS4**	**4.9**	**1.1**	**5.2**	**3.5**	**3.7**	**1.9**	**351.3**	**>400**	**>400**	**>384**	**28.1**	**>103.7**	**71.2**	**>2799.6**	**1443.6**
**MS5**	1.6	0.9	34.7	3.1	10.1	16.4	145.3	>400	30.3	>191.9	189.2	>19.1	88.8	>189.4	5425.6
**MS6**	3.1	4.1	3.6	3.3	3.5	0.4	209.0	99.7	51.9	120.2	80.5	33.9	66.5	956.7	2115.3
**MS7**	**1.6**	**2.8**	**3.3**	**3.0**	**2.7**	**0.7**	**>400**	**>400**	**>400**	**>400**	**0.0**	**>148.7**	**>247.4**	**>5531.3**	**>15304.5**
**MS8**	**2.4**	**3.6**	**4.0**	**3.5**	**3.3**	**0.7**	**>400**	**340.0**	**>400**	**>380**	**34.6**	**>113.5**	**>169.0**	**>3389.4**	**>7141.4**
**MS9**	10.0	50.3	35.0	83.0	44.6	30.5	>400	>400	>400	>400	0.0	>9.0	>40.1	>20.1	>402.7
**MS10**	0.63	0.38	4.63	1.78	1.86	1.95	16.6	120.7	6.7	48.0	63.1	25.9	26.4	1392.4	4190.8
**DXR**	0.22	0.12	0.29	0.09	0.18	0.09	>10	>10	9.2	>9.7	0.4	>54.9	>45.5	>30928.6	>20661.2

Each number is the average of three determinations. Two sets of tumor-selectivity index (TS) and potency-selectivity expression (PSE) values were calculated for all OSCC versus non-malignant cells, as well as paired cells from the same (gingival) tissue. HGF: Human gingival fibroblast; HPLF: Human periodontal ligament fibroblast; HPC: Human pulp cells; CC_50_: 50% cytotoxic concentration; DXR: Doxorubicin; TS: Tumor selectivity index; PSE: Potency-selectivity expression. Ca9-22 are derived from gingival tissue, while HSC-2, HSC-3, and HSC-4 are derived from the tongue.

**Table 2:** Key descriptor properties were identified that significantly impacted cytotoxicity and tumor selectivity (T–N), distinguishing between effects on tumor (T) and normal (N) cells.

	Descriptor	Meaning	Category
**T**	npr2	3D shape and volume	Surface area, volume, and shape descriptors
	E_str	Bond stretch energy	Potential energy descriptors
	SMR_VSA6	Topological shape	Subdivided surface areas
	std_dim2	3D shape	Surface area, volume, and shape descriptors
	SlogP_VSA1	Lipophilicity	Subdivided surface areas
	logS	Lipophilicity	Physical properties
**N**	GCUT_PEOE_2	Topological shape and partial charges	Adjacency and distance matrix descriptors
	E_vdw	van der Waals energy	Potential energy descriptors
	E_strain	Local strain energy	Potential energy descriptors
	PEOE_VSA+2	Topological shape and partial charges	Partial charge descriptors
	PM3_HF	Heat of formation	MOPAC descriptors
	BCUT_PEOE_2	Topological shape and partial charges	Adjacency and distance matrix descriptors
**T-N**	vsurf_R	3D shape	Surface area, volume, and shape descriptors
	a_hyd	Number of hydrophobic atoms	Pharmacophore feature descriptors
	a_nC	Number of carbon atoms	Atom counts and bond counts
	vsurf_D6	3D size and lipophilicity	Surface area, volume, and shape descriptors
	GCUT_SLOGP_3	Topological shape	Adjacency and distance matrix descriptors
	chi0_C	Topological shape	Kier & Hall connectivity and kappa shape indices

**Table 3:** Calculated ADME and physicochemical parameters of the compounds.

Compound	MW (g/mol)	Consensus LogP	HBD	HBA	MR	TPSA (Å^2^)	GI absorption	BBB permeable/penetration (Yes/No)
**MS1**	289.35 g/mol	2.08	2	4	79.72	92.93	High	No
**MS2**	382.44 g/mol	2.60	0	5	103.68	93.01	High	No
**MS3**	327.36 g/mol	1.73	1	5	85.38	103.43	High	No
**MS4**	429.49 g/mol	3.36	1	5	119.80	103.43	High	No
**MS5**	443.52 g/mol	3.82	1	5	124.77	103.43	High	No
**MS6**	459.52 g/mol	3.49	1	6	126.29	112.66	High	No
**MS7**	519.57 g/mol	3.39	1	8	139.28	131.12	Low	No
**MS8**	519.57 g/mol	3.34	1	8	139.28	131.12	Low	No
**MS9**	489.54 g/mol	3.40	1	7	132.78	121.89	Low	No
**MS10**	435.52 g/mol	3.48	1	5	117.68	131.67	Low	No

**Table 4:** Druglikeness properties of the compounds.

Compound	Druglikeness				
	Lipinski	Ghose	Veber	Egan	Muegge
**MS1**	Yes	Yes	Yes	Yes	Yes
**MS2**	Yes	Yes	Yes	Yes	Yes
**MS3**	Yes	Yes	Yes	Yes	Yes
**MS4**	Yes	Yes	Yes	Yes	Yes
**MS5**	Yes	Yes	Yes	Yes	Yes
**MS6**	Yes	Yes	Yes	Yes	Yes
**MS7**	Yes^a^	No^b^	Yes	Yes	Yes
**MS8**	Yes^a^	No^b^	Yes	Yes	Yes
**MS9**	Yes	No^b^	Yes	Yes	Yes
**MS10**	Yes	Yes	Yes	No^c^	Yes

Lipinski filter: MW ≤ 500; MLOGP ≤ 4.15; HBA ≤ 10; HBD ≤ 5

Ghose filter: 160 ≤ MW ≤ 480; – 0.4 ≤ WLOGP ≤ 5.6; 40 ≤ MR ≤ 130; 20 ≤ atoms ≤ 70

Veber filter: Rotatable bonds ≤ 10; TPSA ≤ 140 Å2

Egan filter: WLOGP ≤ 5.88; TPSA ≤ 131.6

Muegge filter: 200 ≤ MW ≤ 600, – 2 ≤ XLOGP ≤ 5; TPSA ≤ 157; HBA ≤ 10; HBD ≤ 5; RB ≤ 15; Number of rings ≤ 7; Number of carbons > 4; Number of heteroatoms > 1

a 1 violation: MW > 500,

b 2 violations: MW > 480, MR > 130,

c 1 violation: TPSA > 131.6

## Footnotes

1 JMP (2024). Statistical Discovery Software [online]. Website: https://www.jmp.com/en_us/home.html [accessed 13 October 2025].
