# Supplementary material for: Tumor-specific cytotoxicity of pyrazole-based chalcone derivatives in human oral squamous cell carcinoma cell lines
Source: Turk J Biol. 2025 Sep 10;49(6):712–27. doi: 10.55730/1300-0152.2773 (PMC12604929; doi:10.55730/1300-0152.2773)

**Tumor-Specific Cytotoxicity of Pyrazole-Based Chalcone Derivatives in Human Oral Squamous Cell Carcinoma Cell Lines**

Mehtap TUGRAK SAKARYA^1^*, Halise Inci GUL^2^, Hiroshi SAKAGAMI^3^, Junko NAGAI^4^, Yoshihiro UESAWA, Kenjiro BANDOW^5^

^1^Department of Pharmaceutical Chemistry, Faculty of Pharmacy, Tokat Gaziosmanpasa University, Tokat, Turkey

^2^Department of Pharmaceutical Chemistry, Faculty of Pharmacy, Ataturk University, Erzurum, Turkey

^3^Meikai University Research Institute of Odontology (M-RIO), Saitama, Japan

^4^Department of Medical Molecular Informatics, Meiji Pharmaceutical University, Tokyo, Japan

^5^Divisions of Biochemistry, Meikai University School of Dentistry, Saitama, Japan

***Correspondence:** [mehtaptugrak@gmail.com](mailto:mehtaptugrak@gmail.com)

ORCIDs:

First AUTHOR: <https://orcid.org/0000-0002-6535-6580>

Second AUTHOR: <https://orcid.org/0000-0001-6164-9602>

Third AUTHOR: [https://orcid.org/0000-0001-8001-2121](https://orcid.org/0000-0002-6535-6580)

Fourth AUTHOR: [https://orcid.org/0000-0002-9069-5752](https://orcid.org/0000-0002-6535-6580)

Fifth AUTHOR: [https://orcid.org/0000-0002-5773-991X](https://orcid.org/0000-0002-6535-6580)

Sixth AUTHOR: [https://orcid.org/0000-0002-1362-3034](https://orcid.org/0000-0002-6535-6580)

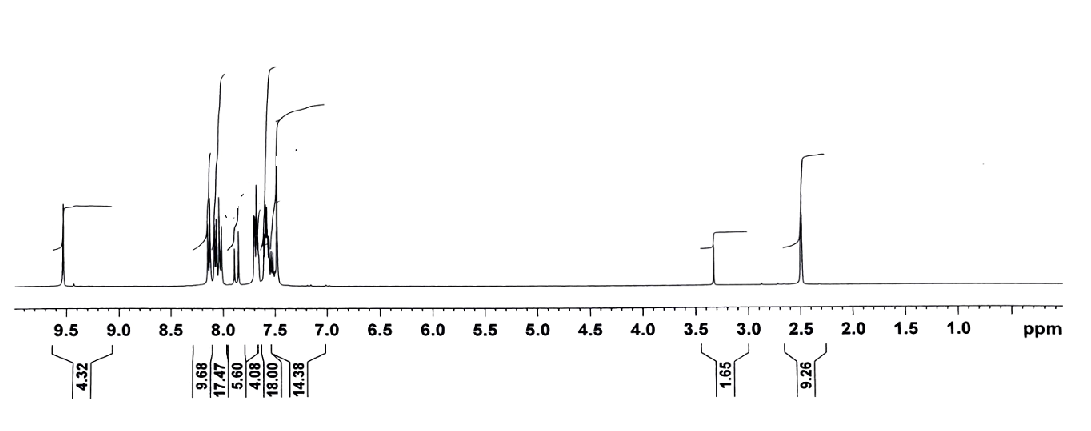
1H NMR

13C NMR


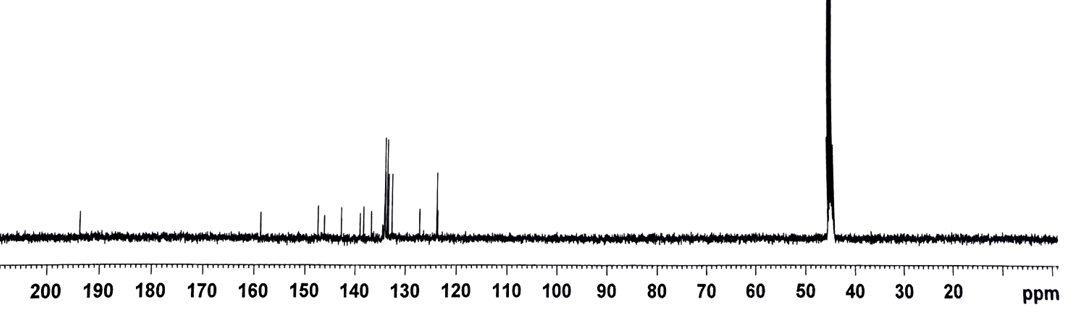


HRMS


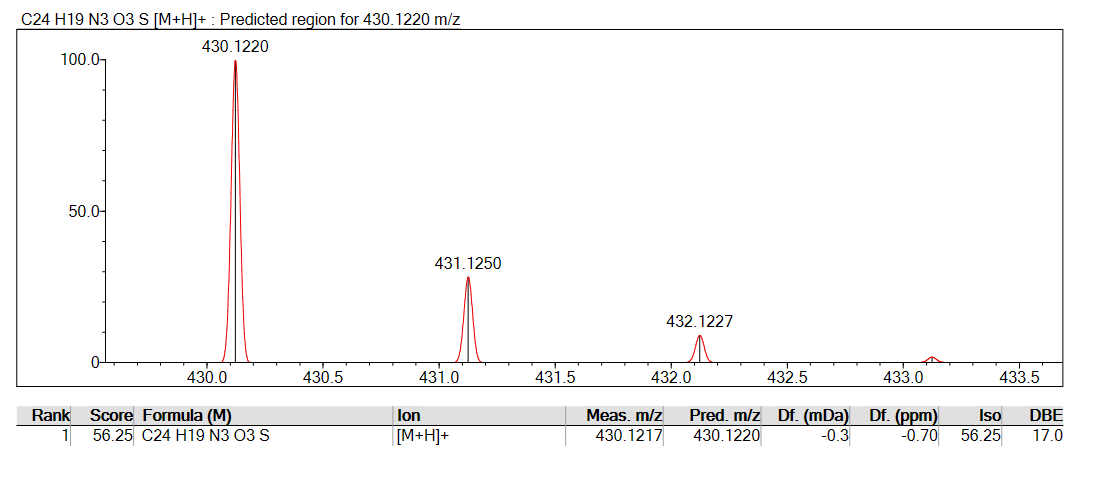

1H NMR


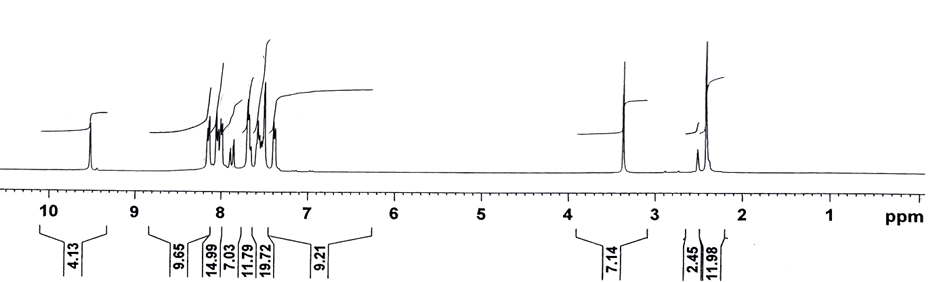


13C NMR


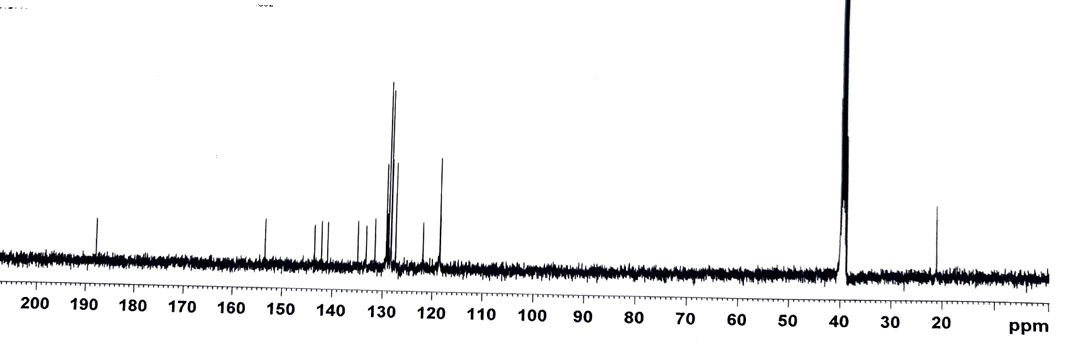


HRMS


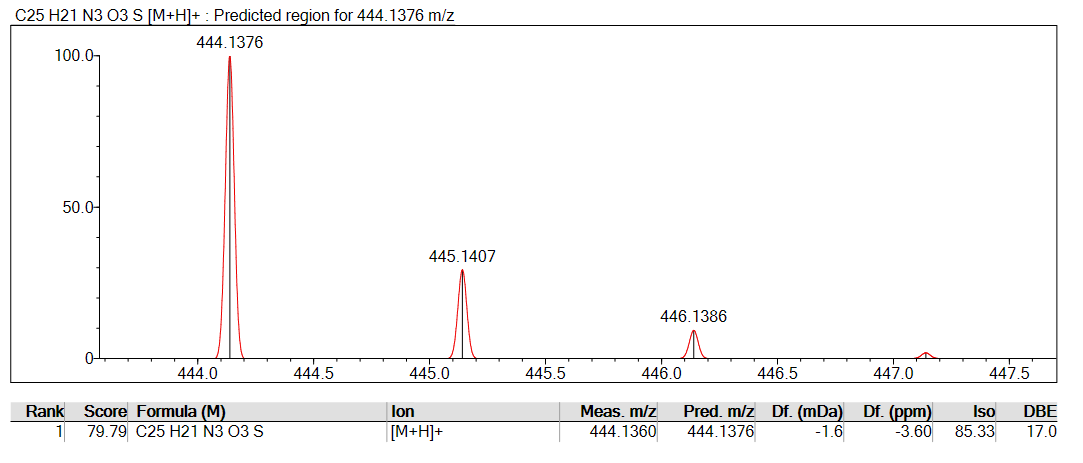

1H NMR
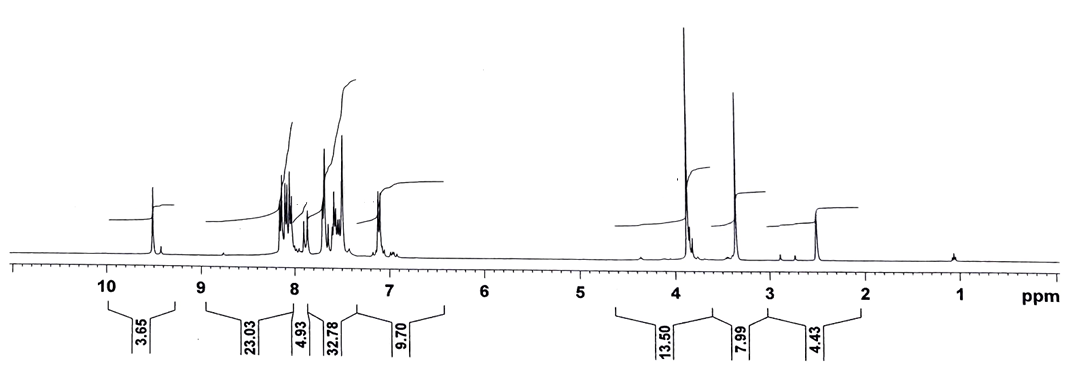


13C NMR


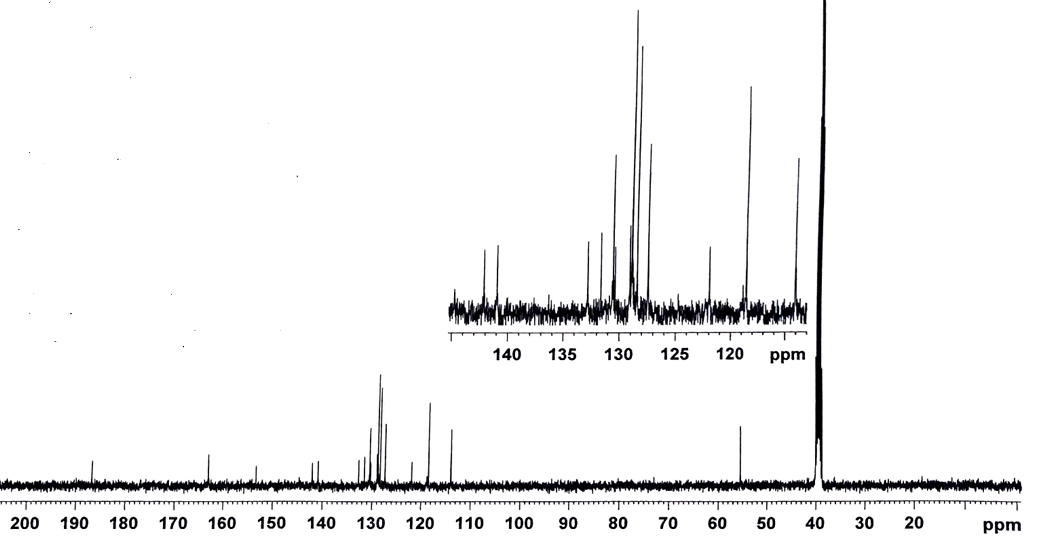


HRMS


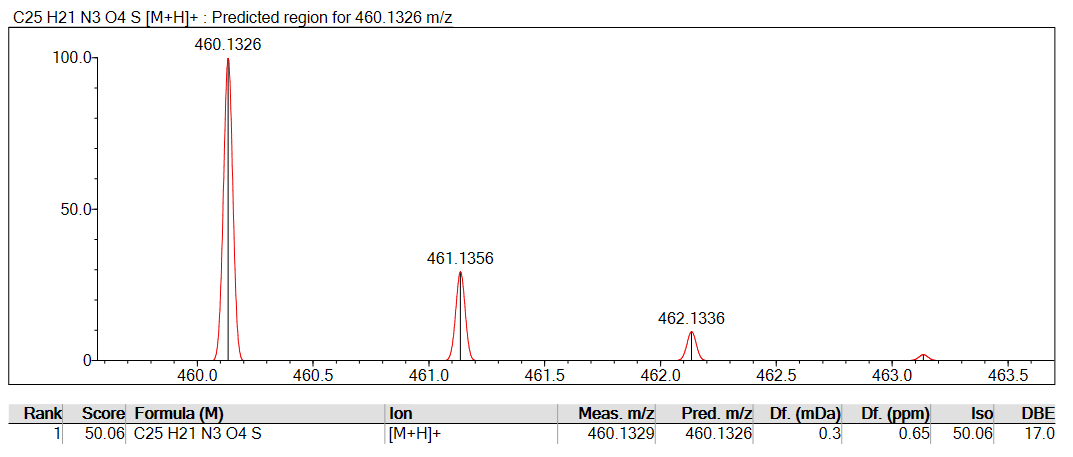

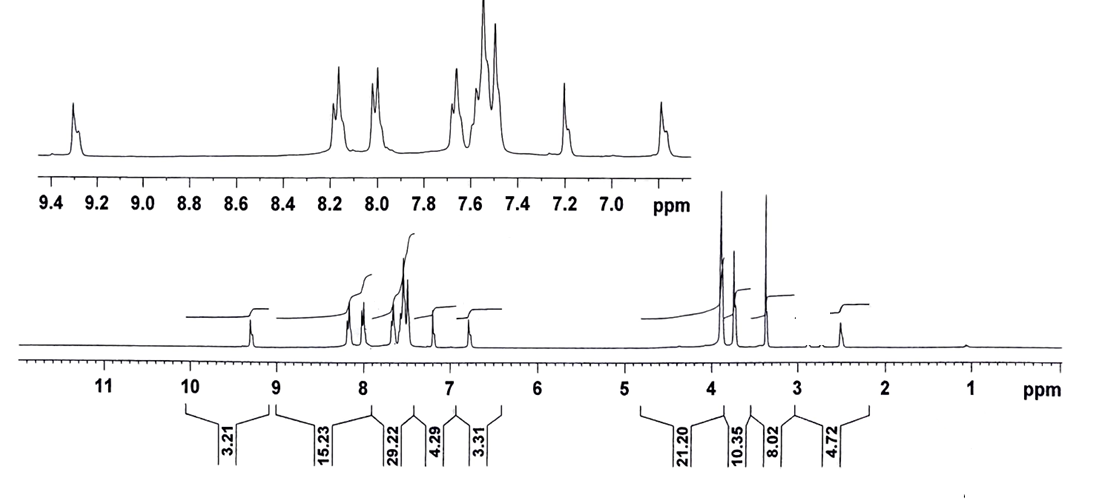
1H NMR

13C NMR


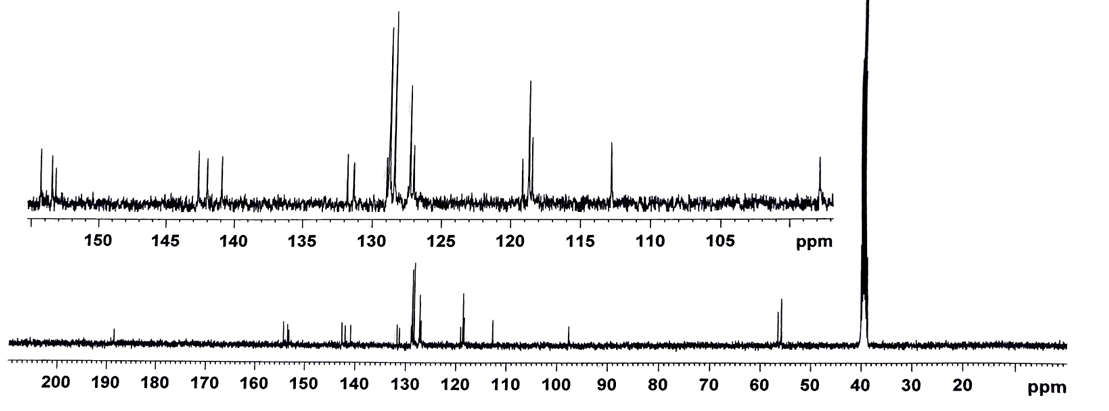


HRMS


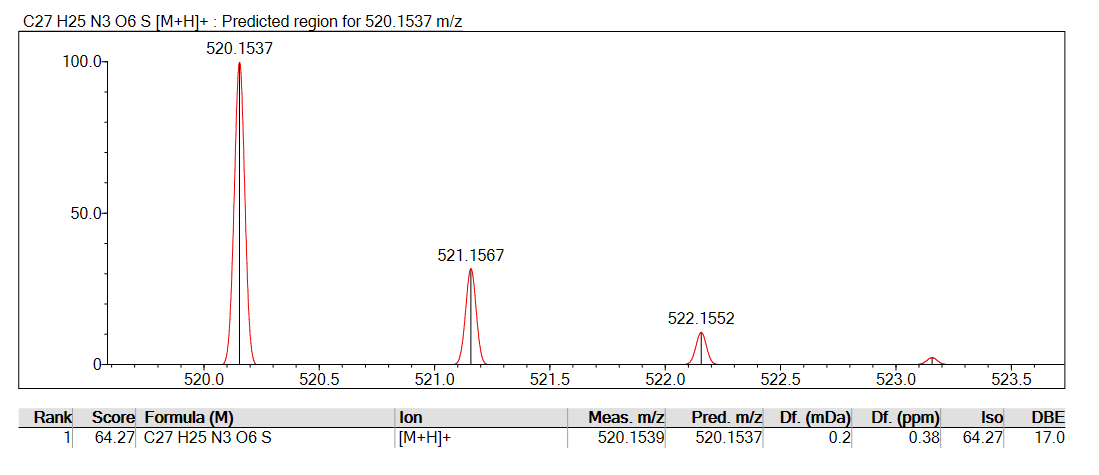

1H NMR


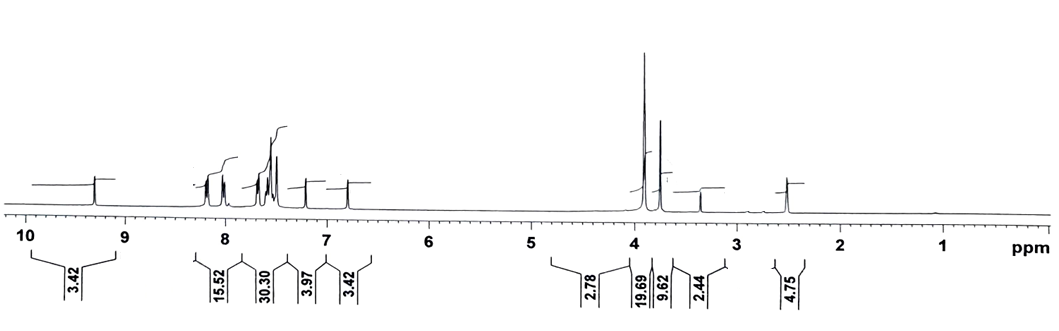


13C NMR


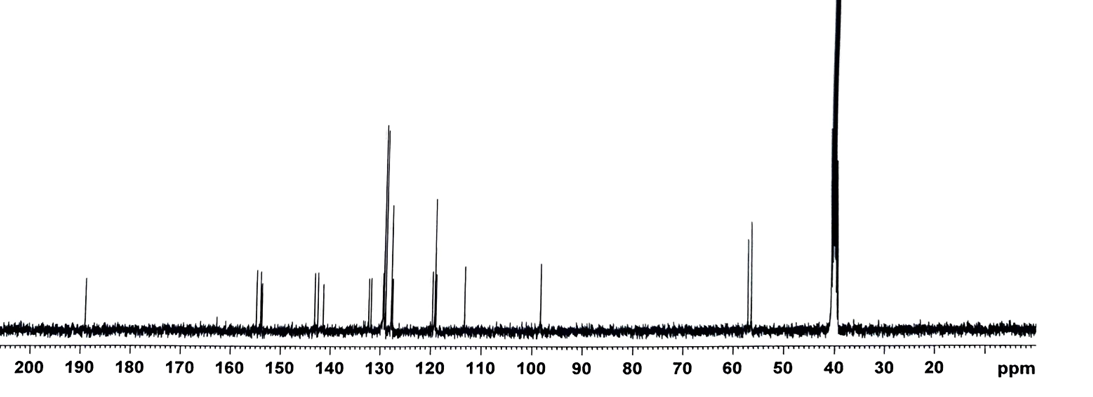


HRMS


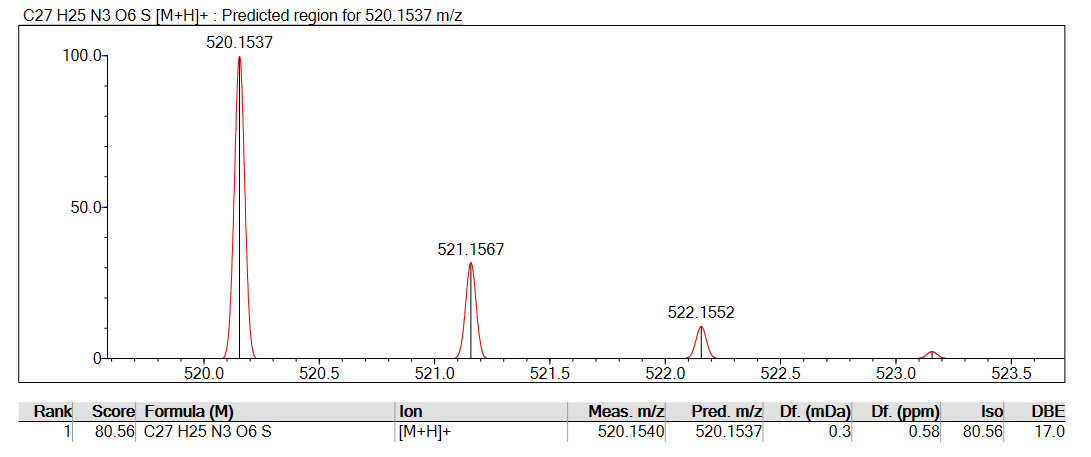

1H NMR


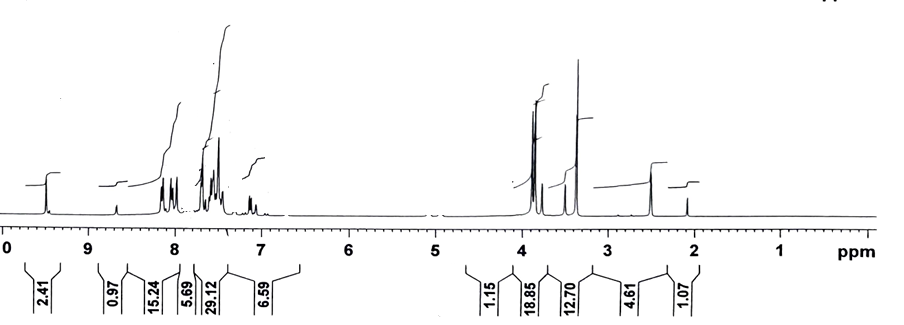


13C NMR


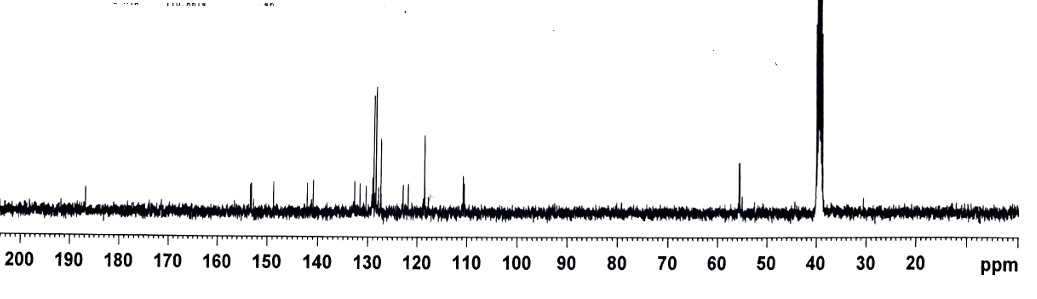


HRMS


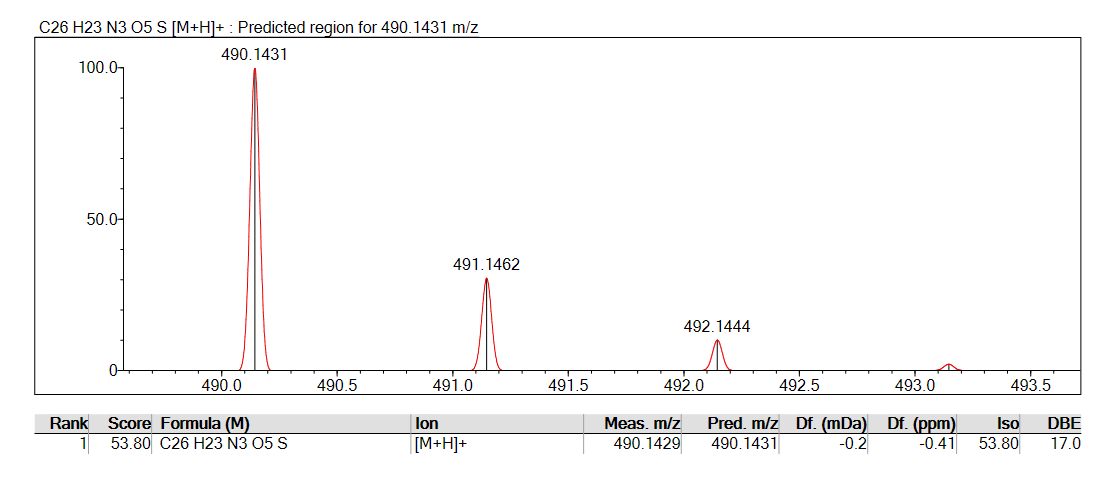

1H NMR


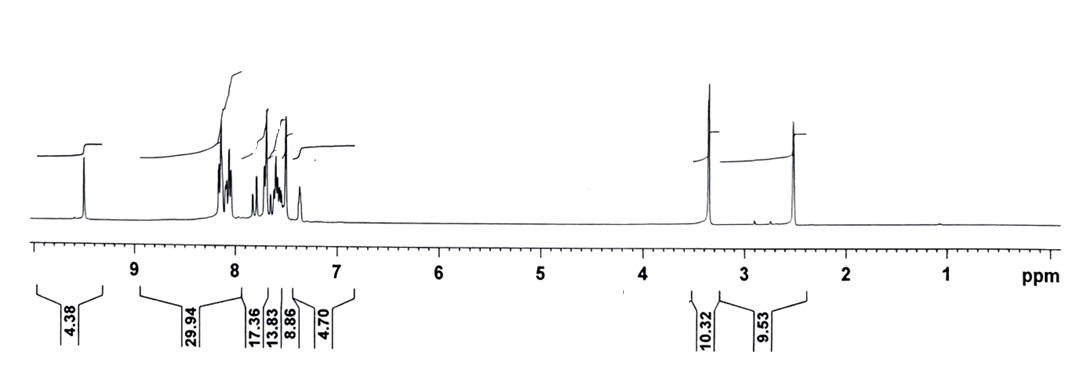


13C NMR


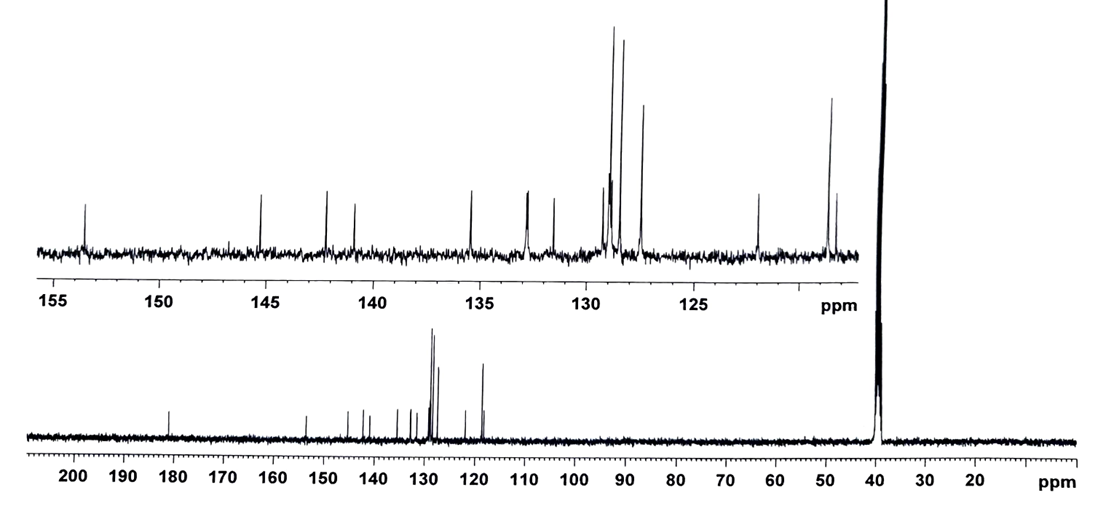


HRMS


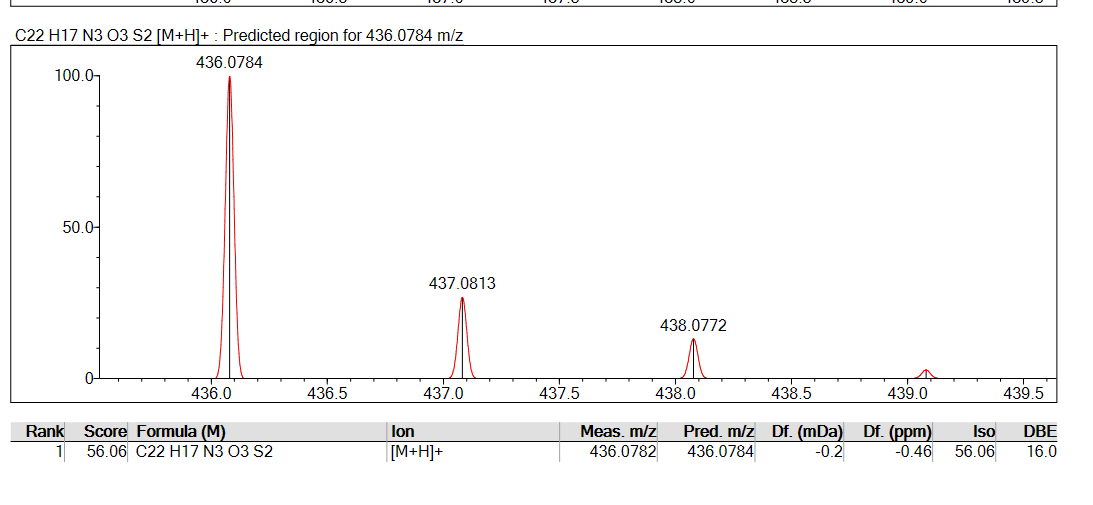

Supplement: Supplementary file 1 [file tjb-49-06-712-Supplementary_file_1.docx]
