# Supplementary material for: Tumor-specific cytotoxicity of pyrazole-based chalcone derivatives in human oral squamous cell carcinoma cell lines
Source: Turk J Biol. 2025 Sep 10;49(6):712–27. doi: 10.55730/1300-0152.2773 (PMC12604929; doi:10.55730/1300-0152.2773)
Supplement: Supplementary file 5 [file Supplemantary_file_4_Material_and_methods_(1).docx]

Material and methods

Assay for cytotoxic activity

Cells were inoculated at 2×103 cells/0.1 ml in a 96-microwell plate. After 48 h, the medium was replaced with 0.1 ml of fresh medium containing MS1~MS10 (0.2, 0.6, 1.3, 2.5, 3.1, 6.3, 13, 25, 50, 100, 200, 400 μM), doxorubicin (0.08, 0.16, 0.31, 0.63, 1.3, 2.5, 5, 10 μM) or 5-FU (8, 16, 31, 63, 125, 250, 500, 1000 μM). Cells were incubated further for 48 h and the relative viable cell number was then determined by the MTT method and expressed as the absorbance of the cell lysate at 560 nm, using a microplate reader (Infinite F50R; TECAN, Männedorf, Switzerland) [49, 50]. Control cells were treated with the same amounts of DMSO and the cell damage induced by DMSO was subtracted from that induced by test agents. The concentration of compound that reduced the viable cell number by 50% (CC_50_) was determined from the dose–response curve and the mean value of CC_50_ for each cell type was calculated from triplicate assays.

Calculation of tumor-selectivity index (TS)

TS was calculated using the following equation: TS=mean CC_50_ against three normal oral cell types/mean CC50 against four OSCC cell lines (HGF, HPLF, HPC vs. Ca9-22, HSC-2, HSC-3, HSC-4). Since both Ca9-22 and HGF cells were derived from the gingival tissue [50], the relative sensitivity of these cells was also compared (as: CC_50_ against HGF/ CC_50_ against Ca9-22).

Calculation of potency-selectivity expression (PSE)

PSE was calculated by the following equation: PSE=mean CC_50_ against three normal oral cell types/(CC_50_ against four OSCC cell lines)2 × 100 (HGF, HPLF, HPC vs. Ca9-22, HSC-2, HSC-3, HSC-4); and as CC_50_ against HGF/(CC_50_ against Ca9-22)2 × 100 using the pair of cell types from the same tissue (gingiva) (see Table 1).

Cell Culture Assay

Human normal oral mesenchymal cells (human gingival fibroblast, HGF; human periodontal ligament fibroblast, HPLF; and human pulp cells, HPC) were established from the first premolar tooth extracted from the lower jaw of a 12-year-old girl [51], and cells at 10-18 population-doubling levels were used in this study. Human oral squamous cell carcinoma (OSCC) cell lines [Ca9-22 (derived from gingival tissue); and HSC-2, HSC-3, HSC-4 (derived from tongue)] were purchased from Riken Cell Bank (Tsukuba, Japan). All of these cells were cultured at 37°C in DMEM supplemented with 10% heat-inactivated fetal bovine serum, 100 units/ml, penicillin G and 100 µg/ml streptomycin sulfate under a humidified 5% CO_2_ atmosphere. Cell morphology was checked periodically under a light microscope (EVOS FL; Thermo Fisher Scientific, Waltham, MA, USA).

Cell-cycle analysis

Treated and untreated cells (approximately 106 cells) were harvested, fixed with 1% paraformaldehyde in PBS without calcium and magnesium ions [PBS(−)]. Fixed cells were then washed twice with PBS(−) and treated for 30 min with 400 μl of 0.2 mg/ml RNase A (preheated for 10 min at 100oC to inactivate DNase) to degrade RNA. Cells were then washed twice with PBS(−) and stained for 15 min with 0.01% propidium iodide (PI) in the presence of 0.01% NP-40 in PBS(−) to prevent cell aggregation. After filtering through Falcon® cell strainers (40 μM) (Corning, NY, USA) to remove aggregated cells, PI-stained cells were subjected to cell sorting (SH800 Series; SONY Imaging Products and Solutions Inc., Kanagawa, Japan). Cell-cycle analysis was performed with Cell Sorter Software version 2.1.2. (SONY Imaging Products and Solution Inc.).

Computational analysis

Estimation of CC_50_ values. Since the CC_50_ values had a distribution pattern close to a logarithmically normal distribution, we used the negative log CC_50_ (pCC_50_) values for the comparison of cytotoxicity between compounds. The mean pCC_50_ values for normal cells and tumor cell lines were defined as N and T, respectively.

Calculation of chemical descriptors. The 3D structure of each chemical structure (MarvinSketch 18.10.0, ChemAxon, Budapest, Hungary) (http://www.chemaxon.com) was optimized by CORINA Classic (Molecular Networks GmbH, Nürnberg, Germany) (https://www.mn-am.com/products/corina) with forcefield calculations (amber-10: EHT) in Molecular Operating Environment (MOE) version 2019.0101 (Chemical Computing Group Inc., Quebec, Canada)(http://www.chemcomp.com/MOE-Cheminformatics_and_QSAR. html). The number of structural descriptors calculated from MOE was 354. Among them, the number of descriptors used for analysis was 286.

Statistical treatment

The CC_50_ values were expressed as mean ± S.D. of triplicate assays. The relation among cytotoxicity, tumor specificity index and chemical descriptors were investigated using simple regression analyses by JMP®Pro version 14.3.0 (SAS Institute Inc., Cary, NC, USA). The significance level was set at p<0.05 (https://www.jmp.com/en_us/home.html). For multiple comparisons, one-way analysis of variance (ANOVA) followed by Bonferroni’s post hoc test was performed (IBM SPSS Statistics version 27.0). A value of p<0.05 was considered to indicate statistically significant.
